# DNA vaccines that express the MPER of HIV-1 gp41 elicit different antibodies depending upon their transmembrane and cytoplasmic domains

**DOI:** 10.1186/1742-4690-9-S2-P348

**Published:** 2012-09-13

**Authors:** JK Scott, N Gulzar, K Klaric, TM Ross, S Lu, S Wang

**Affiliations:** 1 Simon Fraser University, Burnaby, Canada; 2 University of Pittsburgh, Pittsburgh, PA, USA; 3 University of Massachusetts Medical School, Worcester, MA, USA

## Background

The HIV-1 gp41 MPER contains the epitopes for 4 broadly neutralizing (bNt) antibodies (Abs), making it a target for vaccine design. We developed a panel of DNA-vaccine candidates that link the MPER, or larger portions of gp41’s external domain, to two different transmembrane (TM) and cytoplasmic (CT) domains. We report on the ability of DNA vaccines expressing these constructs to elicit MPER-specific Nt Abs in rabbits.

## Methods

DNA vaccines encoding various gp41 ectodomain fragments, and the TM and CT of either the platelet-derived growth factor receptor (PGDFR-TM), or the TM and truncated CT of gp41 (TM1), were used to immunize rabbits; immune sera were tested for reactivity against the MPER displayed on the cell surface and for pseudovirus neutralization.

## Results

Immunizations with plasmids expressing larger fragments of the gp41 ectodomain tethered to the PDGFR-TM elicited lower-titer Abs against the MPER, as compared to the MPER tethered to the PDGFR-TM, which elicited MPER-specific Abs that included those targeting the 2F5 epitope Affinity purification of these Abs on 2F5-epitope peptide resulted in Abs that bound MPER expressed on cells, but neutralized both pseudoviruses bearing HIV envelope and those bearing AMLV envelope. Immunization with DNA vaccines encoding the MPER fused to the gp41 TM1, elicited low-titre Abs that cross-reacted weakly with the MPER and strongly with regions in the CT. All immunization failed to produce Abs that cross-react with the 4E10 epitope.

## Conclusion

While the initial immunization studies reported here demonstrate that MPER reactivity is elicited by DNA immunization, the MPER-TM1 construct appears to elicit Abs against epitopes in its 27-aa CT domain. Current work is focused on replacing the gp41 TM with an engineered TM that will optimally expose the epitopes for the bNt MAbs, particularly 4E10.

